# Hormonal influence on maize inflorescence development and reproduction

**DOI:** 10.1007/s00497-024-00510-0

**Published:** 2024-10-05

**Authors:** Amina Chaudhry, Zongliang Chen, Andrea Gallavotti

**Affiliations:** 1 Waksman Institute of Microbiology, Rutgers University, Piscataway, NJ 08854-8020 USA; 2 Department of Plant Biology, Rutgers University, New Brunswick, NJ 08901 USA

**Keywords:** Maize, Hormones, Sexual reproduction, Plant development

## Abstract

**Key message:**

Different plant hormones contribute to maize reproductive success.

**Abstract:**

Maize is a major crop species and significantly contributes directly and indirectly to human calorie uptake. Its success can be mainly attributed to its unisexual inflorescences, the tassel and the ear, whose formation is regulated by complex genetic and hormonal networks, and is influenced by environmental cues such as temperature, and nutrient and water availability. Traditional genetic analysis of classic developmental mutants, together with new molecular approaches, have shed light on many crucial aspects of maize reproductive development including the influence that phytohormones exert on key developmental steps leading to successful reproduction and seed yield. Here we will review both historical and recent findings concerning the main roles that phytohormones play in maize reproductive development, from the commitment to reproductive development to sexual reproduction.

## Introduction

Plant development proceeds through a constant repetition and maturation of a basic structural unit called phytomer, composed of an internode with a node on one end, a leaf arising from the node, and an axillary meristem in the axil of the leaf. Several internal and external cues guide the progression of phytomers through different stages of development. The life cycle of maize can be roughly divided into three phases: first, after germination the juvenile vegetative phase is denoted by a robust period of growth in terms of vegetative organ initiation such as leaves, internodes, and axillary buds. Second, the adult vegetative phase harbors maturation of phytomers. Finally, the reproductive phase which comprises acquiring reproductive competence by inflorescence development, flowering, and seed production. A hallmark of adult vegetative-to-reproductive transitioning is a change in the identity of meristems. A meristem is a group of undifferentiated, self-replenishing cells responsible for growth, regeneration, and formation of all structures. The shoot of maize arises from the shoot apical meristem (SAM), and when the plant starts to transition, the SAM is determined to form an inflorescence meristem (IM) and abandons further phytomer initiation, channeling its resources into developing inflorescences which will eventually produce seeds for propagation.

**Fig. 1:**
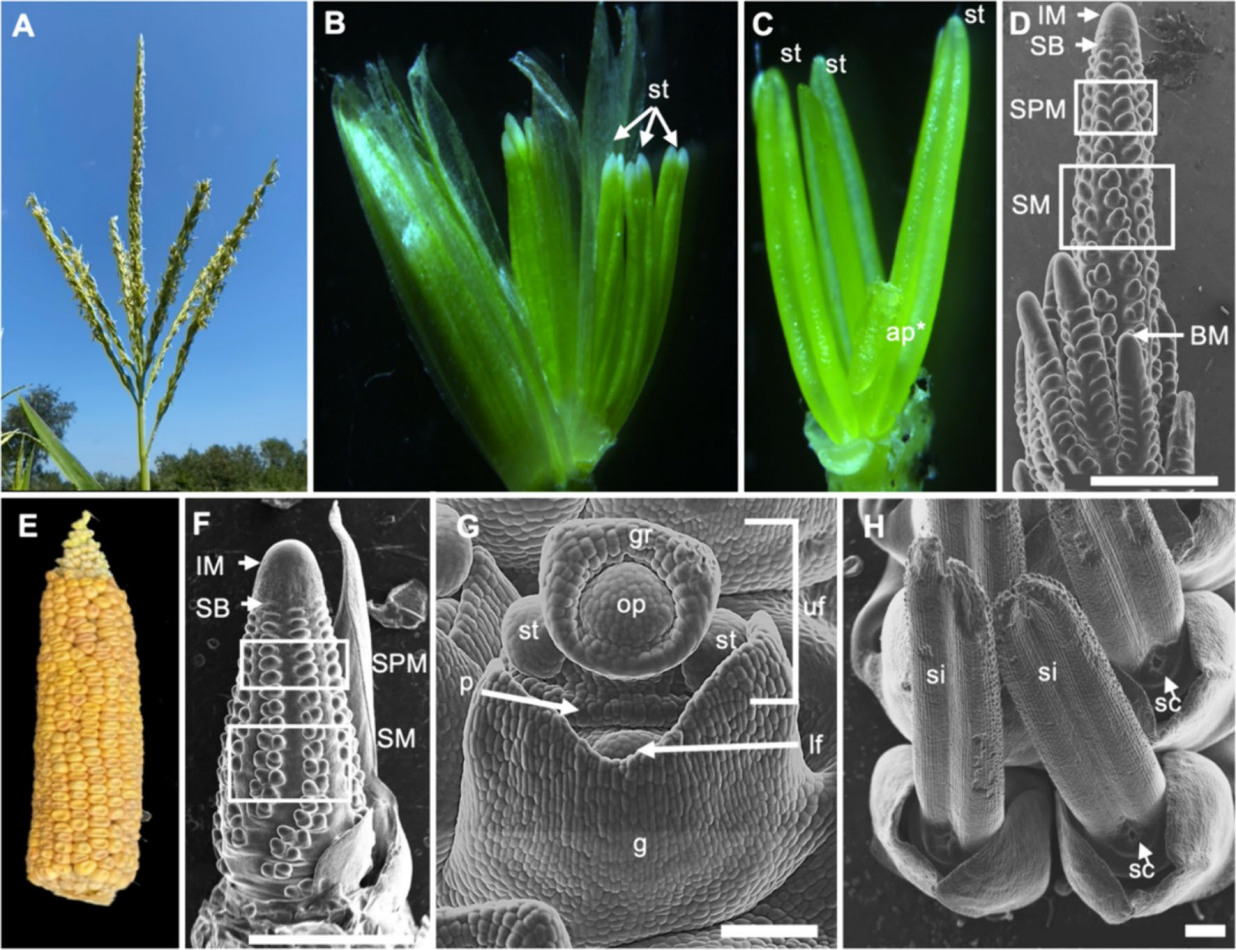
Maize inflorescences showing mature and immature tassel (**A-D**) and ear (**E–H**). **B, C** Dissected mature tassel florets with 3 stamens each. **D**, **F–H** Scanning electron microscopy (SEM) images. **G** SEM of a floral meristem showing the emergence of the gynoecial ridge that will develop into a silk. **H** Emerging silks in ear spikelets. IM: inflorescence meristem; SB: suppressed bract; SPM: spikelet pair meristem; SM: spikelet meristem; BM: branch meristem; st: stamen; ap: aborted pistil; si: silk; sc: stylar canal; pi: pistil; g: glume; gr: gynoecial ridge; op: ovule primordium; uf: upper floret; lf: lower floret; p: palea. Scale bars: 1 mm (**D**, **F**); 100 µm (**G**, **H**)

The staminate inflorescence, the tassel, originates from the SAM at the plant apex and contains a central spike—called rachis—and a variable number of long lateral branches (Fig. 1A–C). Pistillate inflorescences, the ears, are borne laterally in the axil of leaves, lack long branches and develop pistillate florets (Fig. 1E–H). One or more ears may arise at the tips of short lateral branches called shanks several nodes below the tassel. While the development of both inflorescences is remarkably similar, whereby the IM proceeds to initiate rows of axillary meristems (AMs), a notable difference in the early stages of development between the two inflorescences is the formation, in the tassel only, of indeterminate meristems called branch meristems (BMs; Fig. 1D), that eventually form long branches observed in mature inflorescences. In both ear and tassel, IMs proceed to initiate many AMs called spikelet pair meristems (SPMs). Each SPM divides into two spikelet meristems (SMs) which produce an upper and a lower floral meristems each. Each maize floret initiates palea, lemma, two lodicules, three stamens, and three fused carpels. Pistil primordia abort in the tassel, while stamen primordia and the lower floret undergo cell-cycle arrest and abortion in the ear (Dellaporta and Calderon-Urrea 1994). As a result, tassel spikelets have two staminate florets in comparison to a single pistillate floret that reaches maturity in each ear spikelet.

**Fig. 2:**
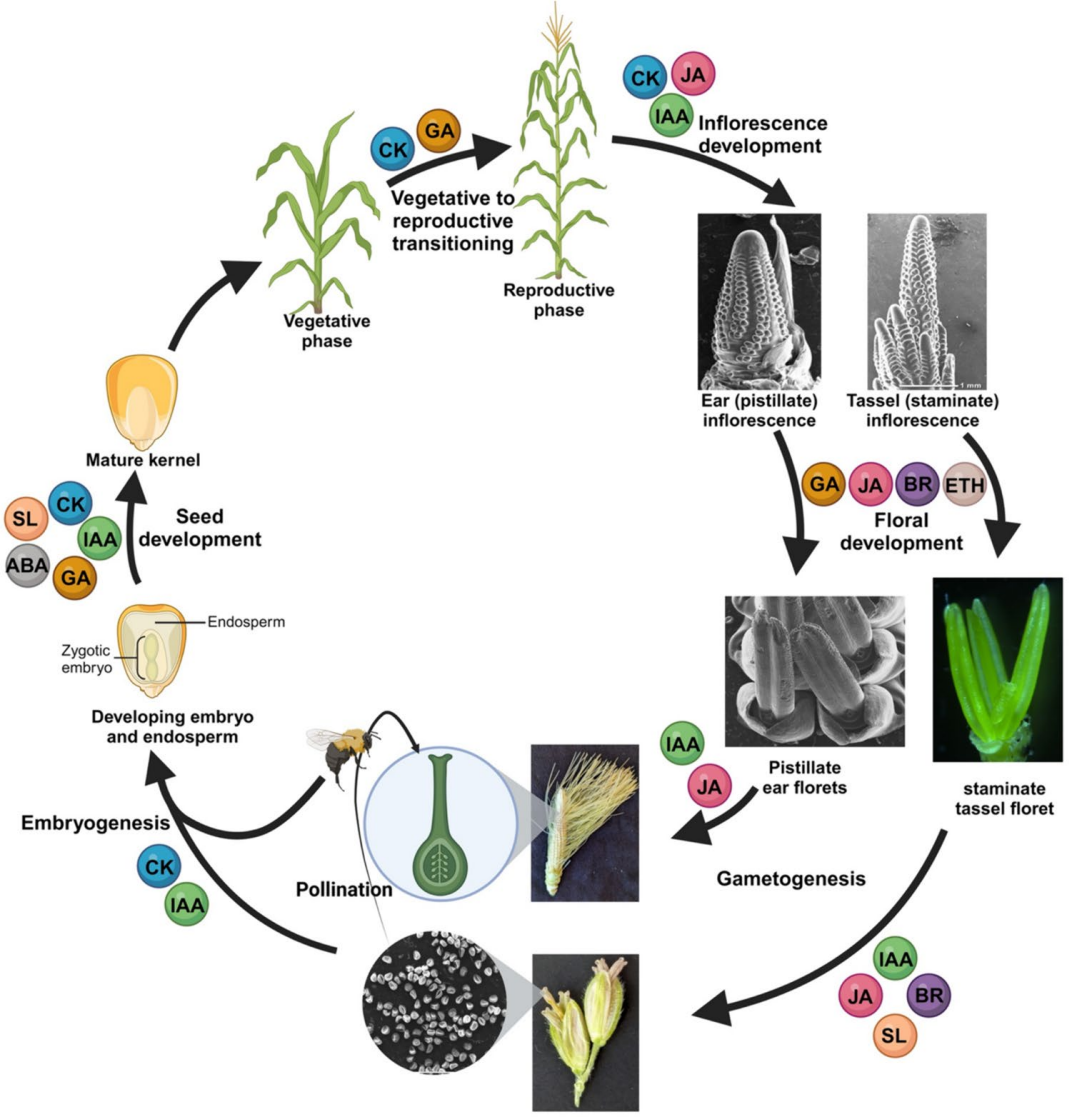
Graphical summary of the known roles of phytohormones in regulating maize inflorescence development and reproduction discussed in this review. Created with BioRender.com

All main phytohormones including auxin, cytokinin (CK), abscisic acid (ABA), gibberellins (GA), brassinosteroids (BR), jasmonic acid (JA), ethylene and strigolactones (SL) play important roles in eventually determining maize inflorescence architecture and reproductive success. These hormones not only have independent roles but also act synergistically to regulate developmental processes from embryogenesis to floral development and seed formation. Here we will review what is known about the hormonal influence of all phases of reproductive development in maize, from the initiation of inflorescences to flowering and sexual reproduction (Fig. 2).

## Vegetative to reproductive transitioning

The transition to flowering represents an important inflection point in the life cycle of Angiosperms, marking the start of their reproductive stage. In maize, the reproductive transition includes the irreversible conversion (determination) of SAM that produces leaves and stem to the tassel IM that develops a compound inflorescence. This critical step is influenced by genetic determinants and environmental cues, such as temperature, photoperiod, and stress.

The phytohormone GA signaling pathway plays a crucial role in enabling informed decision-making by plants regarding the optimal timing for reproductive transition. The GA signaling pathway represents an elegant regulatory mechanism, which involves GA, the GA receptor GIBBERELLIN INSENSITIVE DWARF1 (GID1) and DELLA protein (forming GA-GID1-DELLA module), and it allows plants to flexibly response to endogenous growth trajectories and the exogenous environmental fluctuations (Harberd et al. 2009; Gao et al. 2011). Previous genetic studies on GA biosynthetic genes and blocking of the GA signal transduction pathway have revealed that GA-deficient mutants exhibit a wide spectrum of phenotypes including delayed transitions from the vegetative phase to the reproductive phase, leading to late flowering in maize and Arabidopsis (Evans and Poethig 1995; Plackett et al. 2011), suggesting that GA accelerates flowering time in plants. Among the GAs produced in plants, only a small subset, including GA_1_, GA_3_, GA_4_, and GA_7_, has been demonstrated to function as growth regulators in plant development (Yamaguchi 2008; Hedden and Thomas 2012). Particularly, GA_1_ and GA_4_ are the predominant and active GAs in most plant species. Notably, GA_4_ shows significantly higher bioactivity than GA_1_ in both Arabidopsis and rice, likely due to differences in their binding affinity to the GA receptor, GID1 (Cowling et al. 1998; Ueguchi-Tanaka et al. 2005; Magome et al. 2013; Nomura et al. 2013). The GID1 receptor, initially identified through the characterization of the rice *gid1* mutant, plays a crucial role in GA signaling. The *gid1* mutant exhibits GA-insensitive dwarfism and fails to develop fertile flowers. GID1 is predominantly localized within the nucleus, where it binds specifically to bioactive GAs, initiating downstream signaling pathways that regulate plant growth and flowering (Ueguchi-Tanaka et al. 2005). GA binding to the GID1 receptor enhances the protein–protein interaction between GID1 and a subgroup of the GRAS family of plant-specific transcription regulators, the DELLA proteins, resulting in the rapid degradation of DELLA proteins via the ubiquitin–proteasome pathway mediated by the 26S proteasome (Xue et al. 2022). DELLA proteins act as growth repressors by suppressing the GA response, and their removal releases transcription factors to directly activate downstream genes (Xue et al. 2022). Dominant alleles of the DELLA proteins have been identified across various plant species, and these alleles play a critical role in enhancing crop architecture and have significantly contributed to the improvement of crop traits during the era of “Green Revolution” (Ashikari et al. 2002; Sasaki et al. 2002; Hedden 2003). In maize, the monoecious reproductive trait manifests with specific GA responses in two distinct floral structures: in pistillate ear florets, GA plays a crucial role in suppressing anther formation, while in tassel floretss, an excess of GA leads to the retention of pistils (Bensen et al. 1995; Chen et al. 2014b) (see later section). Possibly due to these unique inflorescence structures and their differential responses to endogenous GA contents, the “Green Revolution” genes associated with GA response weren’t selected for maize improvement similar to other crop species (Thornsberry et al. 2001; Larsson et al. 2013). Indeed, the maize genome contains two characterized paralogous DELLA genes, *DWARF8* (*D8*) and *DWARF9* (*D9*), identified through dominant mutant alleles (Winkler and Freeling 1994; Peng et al. 1999; Lawit et al. 2010). Although quantitative genetic analysis using structured association mapping in maize inbred line populations revealed only weak evidence regarding naturally occurring variation of *D8* associated with maize flowering time (Larsson et al. 2013), the overexpression of a gibberellin-sensitive allele *d9* resulted in earlier flowering time, while overexpression of a gibberellin-insensitive allele *D9-1* caused delayed flowering (Lawit et al. 2010), suggesting that the GA signaling pathway promotes flowering in maize. A recent study investigating transcriptional responses to GA treatments and in dominant alleles of *D8-Mpl* (*D8-Miniplant*) and *D9-1* in maize tassels, provided molecular evidence on how GA regulates early flowering in maize (Best and Dilkes 2022). In this study, the GA_3_ treatment has been found to increase the abundance of *Zea mays FLORICAULA1* (*ZFL1*) and *ZFL2*, two key maize genes play a conserved role in vegetative to reproductive transitioning (Bomblies et al. 2003), as well as the mis-expression of MADS box genes downstream of *ZFL1*/*ZFL2* (Danilevskaya et al. 2008).

Additionally, genes affecting flowering time, such as *CYTOCHROME P450 78A* (*CYP78A*), *PLASTOCHRON1* (*PLA1*), and *GOLDEN-LIKE TRANSCRIPTION FACTOR6* (*GLK6*, a homolog of the Arabidopsis flowering regulator *MYR1*) showed increased abundance after GA_3_ treatments. Conversely, *OUTER CELL LAYERS1* (*OCL1*), known to delay flowering when overexpressed, decreased in expression after GA_3_ treatment. These findings lend additional support to the notion that the GA signaling pathway actively regulates flowering time in maize (Best and Dilkes 2022).

DELLA proteins are widely expressed in leaves and shoot apices, where they interact physically with flowering time-related transcription factors (TFs). *FLOWERING LOCUS T* (*FT*) is involved in one of the key genetic pathways that control flowering time in plants. GA signaling mediated by DELLAs is found to play a significant role in promoting *FT* expression, but it also negatively impacts several *FT* activators, including *CONSTANS* (*CO*). Specifically, DELLAs directly interact with CO through its CCT (CONSTANS, CONSTANS-LIKE, TOC1) domain, preventing CO from binding to the *FT* promoter, leading to lower expression of the *FT* gene, especially in long-day conditions in Arabidopsis (Bao et al. 2020). *FT* homologs have been identified in many plants, including an entire family of *FT*-like genes was named *Zea CENTRORADIALIS (ZCN)* in maize. Particularly, the paralogs *ZCN7* and *ZCN8* have been reported to show florigenic activity (Lazakis et al. 2011). ZCN8 interacts with another TF, DELAYED FLOWERING1 (DLF1)*,* to form a florigen activation complex. *DLF1*, like *ZCN8*, underlies a flowering time QTL in addition to directly activating *ZmMADS4* and *ZmMADS67* which modulate floral transition in the shoot apex (Sun et al. 2020).

Unlike GA, CK prolongs the vegetative phase to ensure that the plant has accumulated sufficient biomass before channeling all resources into reproduction. Known florigen genes in rice are *RICE FLOWERING LOCUS T1* (*RFT1*) and *HEADING DATE 3A* (*HD3A*)*,* which is orthologous to maize *ZCN8* (Park et al. 2008)*.* The expression of both rice florigens is enhanced by the grass-specific type-B RESPONSE REGULATOR (type-B RR) *EARLY HEADING DATE1* (*EHD1*)*,* orthologous to *ZmEHD1* (Brambilla et al. 2017). Exogenous application of synthetic CK, N^6^-benzylaminopurine (BAP), during florigen production delays flowering by suppressing both *HD3A* and *RFT1* expression (Cho et al. 2022). The transcript levels of *ZmEHD1* remained essentially unaltered in *ehd1* knock-out mutants upon CK applications and did not show changes in flowering time when compared to mock samples, suggesting that CKs function in delaying flowering by inhibiting *EHD1*, possibly via physical interaction with type-A ARRs (ARABIDOPSIS RESPONSE REGULATORS), negative regulators of cytokinin signaling (Cho et al. 2022). These studies suggest that excess CK may prolong the vegetative phase by delaying the expression of florigen genes in maize and rice (Cho et al. 2016, 2022). On the other hand, the application of CK to Arabidopsis under short-day conditions has been reported to promote flowering independently of FT but employing its paralog *TWIN SISTER OF FT* (*TSF*)*, FLOWERING LOCUS D* (*FD*)*,* along with *SOC1* (D'Aloia et al. 2011). These contrasting findings suggest that CK regulation is sensitive to temporal developmental context along with environmental cues such as photoperiod.

## Inflorescence development

The early steps in maize inflorescence development include the patterning and specification of new AMs at the peripheral zone of the IM. The first new meristems formed are the SPMs that initiate at the axils of suppressed bract primordia (Fig. 1D, F). In these early stages of development, auxin and cytokinin have a complementary and opposing role in regulating inflorescence meristem function, balancing stem cell proliferation at the central zone of the inflorescence meristem, with new primordia initiation at its peripheral zone. The identification of mutants in auxin biosynthesis, signaling and transport genes affecting these steps highlighted the fundamental role of auxin in organogenesis (Table 1). *SPARSE INFLORESCENCE1* (*SPI1*) encodes a flavin monooxygenase homologous to *YUCCA* (*YUC*) genes in Arabidopsis and catalyzes the rate-limiting step in tryptophan-dependent auxin biosynthesis pathway (Gallavotti et al. 2008a; Zhao 2012). *spi1* mutants are unable to initiate several types of AMs, resulting in the formation of mostly barren tassels with fewer branches and spikelets, and small ears with only a few kernels (Gallavotti et al. 2008a). *SPI1* expression is observed in the peripheral zone of IMs, highlighting the importance of localized auxin burst to drive primordia initiation in inflorescences.

**Table 1:** Maize mutants connected with hormonal regulation of inflorescence development discussed in the text

Maize Mutants	Protein	Hormonal Pathway	References
*Dwarf8 (D8)*	DELLA protein	GA response	Peng et al. (1999)
*Dwarf9 (D9)*	DELLA protein	GA response	Lawit et al. (2010)
*sparse inflorescence1 (spi1)*	YUCCA FLAVIN MONOXYGENASE	TRP-dependent auxin biosynthesis	Gallavotti et al. (2008a, b)
*vanishing tassel2 (vt2)*	TRYPTOPHAN AMINOTRANSFERASE	TRP-dependent auxin biosynthesis	Phillips et al. (2011)
*Barren inflorescence1 (Bif1)*	AUX/IAA	Auxin signaling	Galli et al. (2015)
*Barren inflorescence4 (Bif4)*	AUX/IAA	Auxin signaling	Galli et al. (2015)
*barren stalk1 (ba1)*	bHLH TF	Downstream of auxin signaling	Gallavotti et al. (2004) Galli et al. (2015)
*barren inflorescence2 (bif2)*	SER/THR KINASE	Regulates auxin transport and response	McSteen et al. (2007) Skirpan et al. (2008) Skirpan et al. (2009)
*needle1 (ndl1)*	FTSH METALLOPROTEASE	Indirect effect on auxin homeostasis	Liu et al. (2019a, b)
*Barren inflorescence3 (Bif3)*	WUSCHEL HOMEOBOX TF	CK response	Chen et al. (2021) Chen et al. (2024)
*unbranched3* (*ub3*)	SBP TF	Regulates CK homeostasis	Chuck et al. (2010) Du et al. (2017)
*nana plant1 (na1)*	5α-REDUCTASE	BR biosynthesis	Hartwig et al. (2011)
*nana plant2 (na2)*	Ɗ 24-STEROL REDUCTASE	BR biosynthesis	Best et al. (2016)
*brassinosteroid-deficient dwarf1* (*brd1*)	C-6 OXIDASE	BR biosynthesis	Makarevitch et al. (2012)
*anther ear1 (an1)*	KAURENE SYNTHASE A	GA biosynthesis	Bensen et al. (1995)
*tasselseed1 (ts1)*	LIPOXYGENASE	JA biosynthesis	Acosta et al. (2009)
*tasselseed2 (ts2)*	HYDROXYSTEROID DEHYDROGENASE	JA biosynthesis	DeLong et al. (1993)
*Tasselseed5 (Ts5)*	CYTOCHROME P450 94B1	JA inactivation	Lunde et al. (2019)
*silkless1 (sk1)*	UDP -GLYCOSYLTRANSFERASE	JA inactivation	Hayward et al. (2016)
*defective endosperm18* (*de18*)	YUCCA FLAVIN MONOXYGENASE	TRP-dependent auxin biosynthesis	Bernardi et al. (2019)
*miniature1 (mn1)*	INCW2 CELL WALL INVERTASE	Effects on IAA, CK, JA, ABA and salicylic acid	LeClere et al. (2008) Rijavec et al. (2009)
*auxin response factor17 (arftf17)*	ARF TF	Auxin signaling	Wang et al. (2024)

At the molecular level, the homeobox transcription factor KNOTTED1 (KN1), specifically expressed in meristematic cells, was shown to negatively regulate the accumulation of GA (Bolduc and Hake 2009) and to directly bind to the promoter regions of several genes involved in auxin synthesis, transport, and signaling (Bolduc et al. 2012). KN1 directly binds and negatively regulates *VANISHING TASSEL2* (*VT2*) (Bolduc et al. 2012) which is a co-ortholog of *TRYPTOPHAN AMINOTRANSFERASE OF ARABIDOPSIS1* (*TAA1*), involved in the tryptophan-dependent auxin biosynthesis pathway (Phillips et al. 2011; Zhao 2012). Depleted auxin levels in *vt2* plants cause AM formation defects as well as impaired cell elongation, leading to the formation of short tassels with nearly no functional spikelets or lateral branches. Ears are also negatively impacted and have barren patches, with small sparse kernels (Phillips et al. 2011). VT2 and SPI1 are proposed to work sequentially in the main auxin biosynthetic pathway from tryptophan to indole-3-acetic acid during maize development, in analogous fashion to Arabidopsis (Zhao 2012)**.**

Defective AM initiation in maize inflorescences is a defining feature of the *barren inflorescence* (*bif*) mutant class, which include the recessive *barren stalk1* (*ba1*) and *bif2* mutants, and the semi-dominant *Bif1* and *Bif4* mutants, and is often a read-out of problems in auxin biology*.* Like *pinformed1* and *pinoid* (*pid*) mutants in Arabidopsis, these mutants share a ‘pin’-like inflorescence phenotype, owing to disrupted auxin signaling or transport. *BA1* encodes a bHLH transcription factor, while *BIF1* and *BIF4* encode Aux/IAA proteins (Gallavotti et al. 2004; Galli et al. 2015). These genes were proposed to work in concert for AM formation (Galli et al. 2015). *BIF2*, a co-ortholog of *PID* in Arabidopsis, regulates auxin distribution by controlling the polar localization of the ZmPIN1a protein via phosphorylation (McSteen et al. 2007; Gallavotti et al. 2008b; Skirpan et al. 2009). It is also an interacting partner of BA1, indicating that it may target it for phosphorylation (Skirpan et al. 2008). How phosphorylation affects BA1 function still remains an open question, and while mechanistically it has not been entirely worked out, it is now well supported that BA1 and its interacting partner BA2, another transcriptional regulator, function to initiate AMs after the auxin-driven phyllotactic pattern is established (Wu and McSteen 2007; Gallavotti et al. 2008b; Galli et al. 2015; Yao et al. 2019). Collectively, these studies show that spatiotemporal regulation of auxin biosynthesis, transport, and signaling is fundamental for IM patterning, marked by the positioning of the suppressed bracts, and subsequent AM initiation. However, it remains to be determined whether dynamic auxin minima are required for AM initiation, as described in other species (Wang et al. 2014a, 2014b), a complicated issue to be resolved because of the physical and molecular overlap between suppressed bract and AM primordia at the peripheral zone of IMs (Chuck et al. 2010). Another mutant with striking resemblance to other barren mutants is *needle1* (*ndl1*). Surprisingly, the *NDL1* gene was shown to encode a mitochondria-localized protease that indirectly maintains auxin homeostasis (Liu et al. 2019b).

Cytokinins are directly involved in the CLAVATA-WUSCHEL (CLV-WUS) negative feedback loop that maintains stem-cell populations. WUS is a homeobox TF that promotes stem cell proliferation. It directly activates its negative regulator *CLV3,* which encodes a pre-propeptide that is processed into a 12 amino acid functional peptide perceived by a series of receptor complexes that restrict *WUS* expression to a subdomain of the central zone, called the organizing center (reviewed by Kitakawa and Jackson, 2019 (Kitagawa and Jackson 2019)). Maize homologs of Arabidopsis *CLAVATA* genes have been characterized and include *THICK TASSEL DWARF1*, a functional ortholog of *CLV1*, *FASCIATED EAR2* (*FEA2*) which is a homolog of *CLV2*, *FEA3*, which encode an LRR-RECEPTOR LIKE protein, and *ZmCLE7*, a *CLV3* homolog (Taguchi-Shiobara et al. 2001; Bommert et al. 2005; Je et al. 2016; Rodriguez-Leal et al. 2019). CKs influence this pathway by suppressing *CLV-*mediated feedback on *WUS* (Lindsay et al. 2006; Gordon et al. 2009) and by directly promoting the binding of type-B ARRs to the *WUS* promoter (Meng et al. 2017; Wang et al. 2017; Zhang et al. 2017; Zubo et al. 2017; Xie et al. 2018). Hypersensitivity to cytokinin was inferred in the recently characterized maize *Bif3* mutant, caused by a tandem duplication of *ZmWUS1*, a co-ortholog of *AtWUS* (Chen et al. 2021). A duplicated copy of the *ZmWUS1* gene contains in its proximal promoter region additional binding sites for type-B RRs, causing the formation of enlarged IMs due to over-proliferation of stem cells. These binding sites were shown to have an additive effect on *ZmWUS1* transcription, directly linking cytokinin responsiveness to meristem size regulation in maize (Chen et al. 2024). The functional domains of these inflorescence meristems are also disrupted and appear peculiarly arranged in concentric circles. This rearrangement of functional domains can explain the defects observed in *Bif3* mutants, such as a reduced number of SPMs, the formation of single SMs (rather than paired) and enlarged suppressed bracts, as well as the lack of elongation of ears, which appear ball-shaped and reduced in size (Chen et al. 2021). It remains to be determined why hypersensitivity to CKs leads to the formation of ball-shaped meristems, but this phenomenon was also observed when constitutively active versions of type-B ARR TFs were over-expressed in Arabidopsis (Xie et al. 2018) or predicted via modeling of *CLV3* expression, which appears to be over-expressed and internalized in *ham1,2,3* triple mutants (Zhou et al. 2018).

CK perception and signaling appear to be positively regulated by *KN1* via genes homologous to *WOODEN LEG*, a CK receptor, and *LONELY GUY* (*LOG*), a cytokinin biosynthesis enzyme (Bolduc et al. 2012). The rice *OsLOG* gene is involved in the maintenance of meristem activity. *LOG* encodes a CK-activating enzyme which catalyzes the last step of CK biosynthesis, rendering it bioactive, and is expressed in the panicle BMs and FMs. *log* mutants exhibit premature termination of IM and BMs resulting in significantly reduced inflorescence branching and lower yield (Kurakawa et al. 2007). However, unlike in rice and Arabidopsis, *ZmLOG7* has been reported to affect vegetative development as well, and the mutant shows a meristem termination phenotype during vegetative development (Knauer et al. 2019). Excessive primary and secondary branching is observed in the inflorescence of *cytokinin oxidase/dehydrogenase* (*ckx*) mutants. *CKX* plays a vital role in the degradation of bioactive CKs and a weak allele of *OsCKX2* lead to increased yield (Ashikari et al. 2005). Similarly in barley, the *CKX3* gene was shown to be under direct regulation of the MADS box protein HvMADS1 and regulate cell division in the meristem, influencing spike architecture and branching (Li et al. 2021). The maize *UNBRANCHED3* (*UB3*) gene encodes an SBP transcription factor that controls the initiation of reproductive lateral primordia, together with its paralog *UB2* (Chuck et al. 2014). A micro-RNA resistant version of *UB3* was introduced in both maize and rice plants to identify direct targets of UB3 regulation, and these plants showed upregulation of CK biosynthesis genes and downregulation of CK degradation and auxin biosynthesis genes (Du et al. 2017, 2020). Overall, these studies show the importance of cytokinin in maintaining meristem activity throughout reproductive development in maize and the potential to modulate cytokinin pathways to increase yield.

After the first series of axillary meristems are formed, these proceed through a carefully orchestrated developmental process to the eventual formation of floral meristems. While inflorescence meristems are markedly affected by deficiency in both auxin and cytokinin, all meristems suffer from such deficiencies. For example, auxin biosynthesis mutants such as *vt2* and *spi1* produce spikelets with fewer floral organs (Gallavotti et al. 2008a; Phillips et al. 2011), and mutants affected in the negative regulation of *WUS* such as *td1* and *fea2* show spikelets with additional florets, due to a general enlargement of meristem size (Taguchi-Shiobara et al. 2001; Bommert et al. 2005). However, in the case of the *Bif3* mutant which overexpresses *ZmWUS1*, the severe rearrangement of functional domains and expression of specific marker genes observed in the IM is not obvious in other AMs (Chen et al. 2021). Whether this is an indirect consequence of their large difference in size, or it is due to intrinsic mechanistic differences between meristem types remains to be determined.

## Floral development

After spikelet formation, the developmental programs of tassel and ear start to diverge and eventually lead to the formation of unisexual inflorescences. Developmental cues from gibberellin (GA), brassinosteroid (BR) and jasmonic acid (JA) signaling pathways are intertwined into the complex FM identity developmental program but these interactions mostly remain to be worked out, in particular at the molecular level. For further read on this topic, we recommend a recent review on sex determination mechanisms in maize (Guerrero-Méndez and Abraham-Juárez 2023). Here we summarize the main findings related to the hormonal regulation of sex determination in tassels and ears.

A mutant in the maize homolog of Arabidopsis *DE-ETIOLATED2* (*DET2*) involved in the BR biosynthetic pathway, called *nana plant1* (*na1*), produces feminized tassels, a phenotype called tasselseed (see below), indicating a unique role of the hormone in masculinizing florets in addition to those shared with plant species with bisexual flowers (e.g. in regulating plant height). Applications of propiconazole, a BR biosynthesis inhibitor, mimicked the *na1* phenotype and the mutant accumulated the substrate of the DET2 enzyme and showed reduced levels of all downstream compounds (Hartwig et al. 2011). Other BR biosynthesis mutants, *nana plant2* (*na2*) and *brd1* showed similar effects on tassels (Makarevitch et al. 2012; Best et al. 2016). Double mutant analysis of *na1* and *na2* mutants with mutants in the GA biosynthesis pathway, revealed a context-dependent genetic interaction with additivity observed for certain traits (plant height) and epistasis for others (tillering). In particular, this analysis showed that GA is required for the tasselseed phenotype in the absence of BR. GA mutants, such as *anther ear1* (*an1*) and *dwarf* (*d*) mutants fail to suppress anther development in ears resulting in the anther ear phenotype, a trait that can directly affect yield (Emerson and Emerson 1922; Bensen et al. 1995). Double mutant analysis indicate that the anther ear phenotype is instead unaffected by the loss of BR (Best et al. 2016), uncoupling the two hormones in ear sexual determination.

A prominent class of mutants exhibiting feminized tassels includes *tasselseed1* (*ts1*) to *Ts6*, a collection of recessive and dominant mutants. While *TS3* remains to be identified, *TS1* and *TS2* are assumed to function in the JA biosynthetic pathway (DeLong et al. 1993; Acosta et al. 2009); *TS4* encodes *mir172,* a microRNA that targets AP2 transcription factors, including a dominant miRNA resistant allele of the *INDETERMINATE SPIKELET1* gene, called *Ts6* (Chuck et al. 2007); and *TS5* encodes an enzyme involved in JA catabolism (Lunde et al. 2019). As the name suggests, these mutants are mainly characterized by a failure to abort gynoecia in male reproductive structures. *TS1* and *TS2* genes encode a plastid-targeted lipoxygenase and a hydroxysteroid dehydrogenases, respectively, both of which appear critical for JA biosynthesis. Collectively, the two genes generate cell death cues to abort pistillate structures in the tassel and promote anther maturation (Acosta et al. 2009). Other JA biosynthesis mutants share a same phenotype (Yan et al. 2012). The *SILKLESS1* (*SK1*) gene was shown to act antagonistically to *TS1* and *TS2.* Genetic analysis showed that *sk1* suppresses the *ts1* and *ts2* tassel phenotype and that *ts1* and *ts2* mutants restore pistil production in *sk1* mutant ears (Irish et al. 1994). *SK1* encodes a UDP-glycosyltransferase involved in JA inactivation by conjugation (Hayward et al. 2016). Higher levels of JA were detected in the *sk1* mutant (Zhao et al. 2018) and conversely, undetectable levels were present in transgenic lines overexpressing *SK1* (Hayward et al. 2016). Overexpression of the *SK1* gene prevented all pistil abortion in tassels and ears, leading to the formation of a severe tasselseed phenotype and double pistils in ear spikelets due to the lack of abortion of one floral meristem. This indicates that higher JA levels are required for pistil abortion (Hayward et al. 2016) in accordance with previous reports from Acosta et al. (2009) that rescued the *ts1* phenotype with exogenous applications of JA. In addition to the lack of silk production, the *sk1-A7110* allele also exhibit reduced tassel branching due to a lack of BMs produced at earlier stages of tassel formation (Zhao et al. 2018). This effect was attributed to the downregulation of *UB2* and *UB3* genes, observed in RNA-seq datasets of *sk1-A7110* mutants; this indicates that jasmonic acid may also play a role in the early stages of inflorescence development.

The most recent evidence suggests a complex relationship among JA, BR and GA and the role of *SK1* in sex determination. Genetic work by Best and Dikes (2023) showed that JA and GA act independently and that crosses between *d1* and *sk1* show additive phenotypes, producing plants with only male florets. On the other hand, in double *na2;ski1* mutants pistils were retained in a subset of tassel florets, indicating that pistil production in tassels caused by BR deficiencies is independent of JA. These plants however lacked pistils in the ear, as seen in single *sk1* mutants, suggesting that a general reduction in BR level cannot restore pistil development. *sk1* mutants were reported to have additional developmental defects, including mild ear fasciation, indicating a role in maintaining stem cell populations, and anther preservation in the ear as well. In future work, it would be interesting to determine how JA inactivation influences meristem size regulation, in relation to the known pathways mentioned previously; however, this phenotype may result from residual genetic background effects (Best and Dilkes 2023). Overall, these results indicate that JA and BR influence on pistil development is independent of each other and differs in both inflorescences. This highlights the complexity of hormonal crosstalks and the importance of carefully evaluating genetic interactions among mutants in various pathways.

In addition, several additional factors have been shown to influence sex determination in maize inflorescences, including the MADS-box genes *SILKY1* (*SI1*), *ZMM16/STERILE TASSEL SILKY EAR1* (*STS1*) and *ZAG3/BEARDED EAR* (*BDE*) (Ambrose et al. 2000; Thompson et al. 2009; Bartlett et al. 2015), the DICER-LIKE1 homolog *FZT* (Thompson et al. 2014), the class I HD-ZIP *GT1* gene (Whipple et al. 2011) and the trehalose-6-phosphate phosphatase gene *RAMOSA3* (*RA3*) (Klein et al. 2022). These genes are involved in meristem identity, floral determinacy and/or organ identity specification and they have not been all directly connected to specific hormonal pathways. In Arabidopsis, however, GA promotes the expression of B and C-class MADS-box genes (Yu et al. 2004); whether this is the case for maize florets needs to be determined. This and other potential interactions need to be properly tested by genetics or by treatments with hormones or specific hormone inhibitors (Best et al. 2017) to establish a more comprehensive view of how hormonal signaling pathways intersect with well-established genetic networks regulating inflorescence and floret development (Eveland et al. 2014). A recent study using scRNA-seq analysis of developing tassels and ears identified regulatory modules connecting hormonal pathways that drive sex differentiation with known players such as RA3, GT1 and SK1, as well as several potential new regulators of pistil suppression in tassels and stamen arrest in ears (Sun et al. 2024).

The gaseous hormone ethylene also plays a crucial role in regulating flower development and sex determination. In maize, genetic manipulations that repress ethylene biosynthesis or response have been shown to enhance maize grain yield under drought and low nitrogen conditions (Habben et al. 2014; Shi et al. 2015, 2017). A recent study discovered that reducing ethylene production in developing maize ears by loss-of-function mutations in the gene underlying *qEL7* (a QTL for ear length), which encodes a 1-AMINOCYCLOPROPANE-1-CARBOXYLATE OXIDASE 2 (ACO2), increased ear length and kernel yield. The *qEL7*/*ACO2* gene functions in the final step of ethylene biosynthesis. The reduction in ethylene production promotes meristem and flower development, as well as floret fertility, resulting in approximately a 13.4% increase in grain yield per hybrid ear, indicating that the *ZmACO2* gene negatively regulates kernel number and grain yield (Ning et al. 2021). Likely, these effects involve cross-talks with other hormonal pathways, especially auxin signaling and auxin and JA biosynthesis, and with genes regulating meristem maintenance and identity (Ning et al. 2021). These findings suggest that a better understanding of hormonal crosstalk during inflorescence and floret development may lead to yield increase in maize and other grasses, especially in less ideal environments.

## Gametogenesis and sexual reproduction

After floral organ specification, the plant proceeds to form male and female gametophytes for sexual reproduction. Maize produces copious amounts of pollen, which is housed in filamentous anthers. The development of the anther can be divided into four distinct phases: (1) archesporial cell specification, (2) anther somatic cell division, (3) tapetum development and pollen mother cell meiosis, and (4) mature pollen formation and anther dehiscence (reviewed by Wan et al. 2019) (Wan et al. 2019).

Analyses of mutants in the JA and auxin pathways in Arabidopsis and rice have provided insights into the hormonal regulation of anther development but much remains to be learned in maize. JA appears to act downstream of auxin to orchestrate anther dehiscence, pollen maturation, and filament elongation (Cecchetti et al. 2008, 2013; Song et al. 2018). Impaired filament elongation and delayed or failed anther dehiscence may result from the misregulation of water content in the stamens which is controlled by JA (Wilson et al. 2011). Several Arabidopsis mutants have been reported to exhibit anther dehiscence defects, including *fatty acid desaturation* (*fad*) mutants, *12-oxophytodienoic acid reductase* (*opr3*) mutant, *delayed-dehiscence1* (*dde1*) and *dde2*, *defective in anther dehiscence 1* (*dad1*), *allene oxide synthase* (*aos*) mutants, and *coronatine insensitive* (*coi1*) mutants (McConn and Browse 1996; Sanders et al. 2000; Stintzi and Browse 2000; Ishiguro et al. 2001; Devoto et al. 2002; Park et al. 2002; von Malek et al. 2002).

Successful pollination and reproduction rely on proper anther dehiscence, with JA playing a crucial role in this process. In rice, OsTIE1, an EAR motif-containing TCP transcription factor predominantly expressed in anthers, tightly regulates JA biosynthesis during anther development by repressing another TCP transcription factor, OsTCP1/PCF5. *Ostie1* mutants are male sterile, produce non-viable pollen, and show early stamen filament elongation and precocious dehiscence. OsTCP1 promotes JA synthesis by activating the expression of JA synthesis genes, including *LIPOXYGENASEs* (*LOXs*), while OsTIE1 interacts with OsTCP1 to suppress the expression of JA synthesis genes, providing a fine-tuning mechanism for generating male fertile plants (Fang et al. 2024). Additionally, the timing of anther dehiscence in rice is regulated by *FT-INTERACTING PROTEIN7* (*OsFTIP7*). *OsFTIP7* expression peaks in anthers before pollen mitotic divisions. OsFTIP7 was found to physically interact with OSH1, the rice KN1 ortholog, and facilitate its nuclear translocation and repression of the auxin biosynthetic gene *OsYUCCA4*, resulting in reduced auxin levels in late stages of anther development. This intricate interaction involving *OsFTIP7*, *OSH1*, and *OsYUCCA4* contributes to the precise regulation of rice anther dehiscence (Song et al. 2018).

Catabolism of free auxins is also necessary for proper anther development. In rice, this is partially accomplished by the DIOXYGENASE FOR AUXIN OXIDATION (DAO) enzyme, and *dao* mutants exhibit high auxin levels that correlate with reduced jasmonic acid and anther indehiscence (Zhao et al. 2013). Maize has two putative co-orthologs, *DAO1* and *DAO2*, that remain uncharacterized. In a recent report in barley, auxin was reported to play a role in shifting metabolism during pollen starch accumulation, a key step for pollen maturation and successful fertilizations thanks to the pollen specific *HvYUCCA4* gene. Maize has three closely related homologs that may play a similar function, but this remains to be explored (Amanda et al. 2022).

During gynoecium development, ovule primordia are initiated from the placental tissue. The absence of placenta or ovules is commonly seen among Arabidopsis mutants in auxin biosynthesis and polar transport pathways, such as *yuc1yuc4* (Cheng et al. 2006) and *pin1-1* (Okada et al. 1991), respectively. Similarly, mutants with inadequate BR levels, namely *brassinosteroid-insensitive 2* (*bin2-1*), *brassinosteroid-insensitive 1* (*bri1-5*), and *det-2* form shorter gynoecia and a limited number of ovules (Huang et al. 2013). On the other hand, *ckx3 ckx5* mutants show enlarged gynoecia and overactive placental meristem that produces twice the number of ovules attributed to compromised degradation and elevated CK levels (Bartrina et al. 2011). An enhanced BR signaling mutant, *brassinazole-resistant 1-1D* (*bzr1-1D*), is also reported to have a *ckx3 ckx5*-like effect resulting in an increased number of ovules (Huang et al. 2013).

Concomitantly, the ovular tissue provides protection and nourishment to the deeply embedded female gametophyte. Precise coordination and communication between the two are required during the developmental stages as the smallest aberration may cause abortion of the ovule and female infertility (Nonomura et al. 2007; Yang et al. 2017; Liu et al. 2019a). Transcriptome analysis performed in rice shows a concerted action of auxin and cytokinin. Particularly, the expression of gene families involved in auxin biosynthesis (*OsYUC*) and transport (*OsPIN*) peaked in the sporophytic ovule tissue at the micropylar end of the nucellus, but this signal was subdued as development progressed. The expression of CK synthesis genes (*OsIPT1* and *OsIPT3*) overlapped with auxin production during the early phase of ovule development. While the sporophyte-gametophyte crosstalk is spatially well-regulated, the upregulation of CK-repressing type-A ARRs suggested negative feedback for temporal control as well (Wu et al. 2015).

Following successful gametophyte development, the plant undergoes sexual reproduction. This involves pollen transfer from the tassel to the receptive ear via silk, leading to double fertilization and the formation of a diploid zygote (Doll et al. 2017). Although this process is complex at the molecular and genetic levels, our understanding of the role of hormones in fertilization is limited. However, a handful of studies have indicated some aspects of hormonal regulation in this process. For example, GA plays a significant role in silk and ovule development (Li and Liu 2017). Additionally, SLs have been shown to have a negative role in silk development in maize. Transgenic maize lines overexpressing an Arabidopsis SL-sequestering enzyme, CARBOXYLESTERASE 20 (AtCVE20), promoted silk growth and reduced the anthesis-silk interval in drought conditions, an important yield trait (Roesler et al. 2021). Moreover, overexpression of the maize SL receptor *ZmD14* in Arabidopsis led to upregulation of ABA biosynthesis and signal transduction genes *NCED3* and *SnRK2*.6. The overexpression lines also showed elevated expression of the *AOS1* gene associated with JA biosynthesis and a decrease in the JA signaling gene *JAZ1*. The study revealed a significant interplay of SLs with JA and ABA pathways (Zhang et al. 2024).

## Embryo and seed development

Contrary to eudicots, a transcriptomic study conducted by Chen et al. (2017) suggested that hormones play a minor role during the initial stages of early embryogenesis in maize. After double fertilization occurs in the maize embryo sac, the zygotic genome becomes activated approximately 12 h after pollination, leading to the maternal-to-zygotic transition. Subsequently, the zygote undergoes asymmetric cell division, resulting in apical cells that give rise to the embryo proper and later mature into the complete embryo, while basal cells develop into the suspensor (Chen et al. 2017). The suspensor, responsible for transferring nutrients from maternal tissue to the developing embryo, is believed to undergo programmed cell death after completing its function approximately 14 days after pollination (DAP) (Giuliani et al. 2002; Consonni et al. 2005). The embryo initially acquires a globular structure with radial symmetry, followed by the lateral organ formation alongside the establishment of pluripotent meristems, where the stem-cell reservoirs give rise to shoot apical meristem and root apical meristem. The first lateral organ of maize embryo is the scutellum, which is a shield-shaped and glandular structure that functions to absorb nutrients from the germinating seed. Subsequently, the coleoptile emerges as the second embryonic structure, forming a cylindrical sheath that protects the shoot during seedling germination. Recent transcriptomic analysis of maize developing embryos, employing advanced techniques such as single-cell RNA-sequencing (sc-RNAseq), spatial transcriptomics, and multiplexed in situ RNA-targeting of core developmental genes, provides support for a model in which the maize cotyledon is a bipartite embryonic organ. Specifically, this cotyledon consists of two distinct domains: the scutellum (a non-foliar leaf) and the coleoptile, and each of these domains serves different functions within the single grass cotyledon (Wu et al. 2024).

A recent high-temporal-resolution transcriptome atlas of maize embryo sac and ovule during early seed development uncovered auxin biosynthesis, transport, and signaling-related genes expressed at different development stages and subregions of the seed (Li et al. 2023). Auxin signaling, detected by monitoring activity of a DR5 reporter, was first observed in the endosperm, but not in the pro-embryo, shortly after fertilization. The DR5 endosperm signal gradually increased and covered the surface of the entire embryo by 8 DAP (days after pollination), with the first instance of auxin response also visualized in the most apical cells of the scutellum (Chen et al. 2014a; Murphy et al. 2022). Similarly, the expression of the CK biosynthesis gene *ISOPENTYL TRANSFERASE2* (*ZmIPT2*) was reported as early as 8 DAP, when the embryo is already composed of hundreds of cells. The expression peaks in the basal transfer cell layer (BETL; a juncture of filial and maternal tissues that serves as an entry point of nutrients into the seed), embryo, and kernel at 10 DAP, coinciding with elevated CK levels, indicating an important role for CK in cell proliferation in the embryo and the endosperm affecting seed size (Brugiere et al. 2003, 2008).

Failure to accumulate auxin in the *ZmYUC1* mutant *defective endosperm 18* (*de18*), results in impaired control of sugar and protein metabolism during kernel differentiation. IAA concentration is also crucial for BETL formation. The expression of the CK biosynthesis gene *ZmIPT2*, and CK degradation enzyme, *CYTOKININ OXIDASE 12* (*CKX12*), was also downregulated in the *de18* mutants (Bernardi et al. 2019). Another integral component of CK signaling are type-A ARRs. A pair of endosperm-specific type-A RRs, *ZmTCRR1* and *2*, were detected in BETL cells from 8 to 14 DAP (Muniz et al. 2006), with *ZmTCRR2* expression being detectable as late as 20 DAP (Muniz et al. 2010). The expression of both genes is regulated by a master regulator of BETL identity, *ZmMRP1* (Royo et al. 2022) and both proteins accumulate in the developing endosperm storage area (Muniz et al. 2006, 2010). By overexpression in Arabidopsis, it was suggested that *ZmTCRR2* and *ZmTCRR1* negatively influence auxin and cytokinin signaling in endosperm cells (Royo et al. 2022).

During maize grain filling, the concentration of free auxin (IAA) increases in the growing endosperm, while it decreases in the developing embryo. This could be due to two reasons: (i) the expanding endosperm cells have a greater need for active, free IAA to promote their growth and differentiation; (ii) the endosperm may be synthesizing and accumulating large storage reserves of IAA-sugar conjugates. This spatial and temporal regulation of auxin homeostasis is crucial for coordinating the growth of these two key maize seed tissues during grain filling (LeClere et al. 2008). Another enzyme localized predominantly in the BETL is the cell wall invertase, INCW2, encoded by the *ZmMn1* (*MINIATURE1*) locus, which converts sucrose into glucose and fructose (LeClere et al. 2008). The *mn1* mutants have defective endosperm development, and consequently having 30% less endosperm weight at maturity (Rijavec et al. 2009). Analysis of *mn1* mutants indicates a pleiotropic effect on several hormonal pathways, including IAA, JA, ABA and salicylic acid (LeClere et al. 2008). A solution of GA has been reported to improve the grain-filling rate of maize by increasing levels of several phytohormones. Contents of CK, IAA, and ABA was positively correlated with grain weight and grain-filling rate of maize (Cui et al. 2020).

Recently, SLs have been shown to be also involved in kernel development, influencing both architectural traits and grain filling (Guan et al. 2023). Similarly, mutations in the clade C *ARFTF17* (*AUXIN RESPONSE FACTOR17*) gene were recently reported to reduce IAA content and affect the shape of maize kernels, creating shorter, denser kernels. Clade C ARFs are the least characterized and understood members of the ARF family of transcriptional regulators, involved in auxin signaling (Galli et al. 2018). This study indicates that ARFTF17 physically interacts with MYB40 and modulates its dual activity as an activator and repressor of transcription, affecting among others the expression of the *ZmPIN1a* transporter. An EMS mutation in *ZmPIN1a* indeed revealed similar effects on kernels (Wang et al. 2024).

## Conclusion

In this review, we presented significant advances that have been made in understanding the main effects of hormones on key developmental steps necessary for successful maize reproduction, and these have been mainly driven by studying classic mutants collected over decades (Nannas and Dawe 2015; Richardson and Hake 2022). These studies have revealed major effects that certain hormones play in specific developmental stages; achieving a more comprehensive picture of hormonal interplay during development though will require a thorough combination of genetic and molecular approaches. Crucial fundamental knowledge is still missing. For instance, a special case of embryogenesis is somatic embryogenesis, the formation of new embryos from differentiated tissue. This process is of particular importance to new approaches to maize (and other monocot) transformation, including those employing morphogenic regulators. In recent years, different systems have been developed to improve maize transformation efficiency based on the expression of specific genes involved in meristem or embryo development, including the maize *WUSCHEL2*, *BABY BOOM* (Lowe et al. 2016; Jones et al. 2019; Wang et al. 2023) and *WOX2a* genes (Liu et al. 2023; McFarland et al. 2023). While it is well established that auxin and cytokinin play a major role in the process and they are both commonly added to tissue culture media, the molecular mechanisms driving this process in maize have not been explored in detail (Chen et al. 2022). This knowledge is essential to understand and eventually overcome some of the remaining obstacles to maize and other monocot transformation, including tissue and genotype dependency.

Fortunately, new tools to help fill these gaps are now rapidly becoming available and accessible to maize researchers. Reverse genetic approaches are now largely productive thanks to CRISPR-Cas9 mediated editing that, unlike traditional mutagenesis, can overcome the issue of redundancy and achieve mutant combinations without the need of lengthy genetic crossing. Tremendous advances in single-cell genomic strategies, both for transcription and chromatin status (Satterlee et al. 2020; Marand et al. 2021; Xu and Jackson 2023), should allow in-depth explorations of how hormonal pathways shape maize development and their interaction with the environment, a crucial knowledge to secure maize as a major food source in future decades.
